# Livestock grazing impact differently on the functional diversity of dung beetles depending on the regional context in subtropical forests

**DOI:** 10.1038/s41598-022-05616-x

**Published:** 2022-01-31

**Authors:** Celeste B. Guerra Alonso, Gustavo A. Zurita, M. Isabel Bellocq

**Affiliations:** 1 Instituto de Biología Subtropical, Universidad Nacional de Misiones-CONICET, Puerto Iguazú, Misiones Argentina; 2 Facultad de Ciencias Forestales, Universidad Nacional de Misiones-CONICET, Eldorado, Misiones Argentina; 3 Departamento de Ecología, Genética y Evolución, Facultad de Ciencias Exactas y Naturales, Universidad de Buenos Aires, Buenos Aires, Argentina

**Keywords:** Ecology, Ecology, Environmental sciences

## Abstract

The replacement of native forest by cattle pastures reduces functional diversity; however, little is known about whether the changes depend on regional variation. Dung beetles are one of the most diverse and functionally important taxa; through organic matter burial, dung beetles improve soil quality. We collected dung beetles in native forests and cattle ranching areas in subtropical forests with contrasting climatic conditions: the Atlantic Forest, the Humid Chaco, and the Dry Chaco. We measured 11 traits related to the ecology and the physiology of species. Irrespectively of the region, functional richness was higher in forests (native and with cattle) when compared to open pastures. Humid forests (Atlantic Forest and Humid Chaco) showed higher functional richness than Dry Chaco. Functional dispersion in humid forests was similar between native forest and livestock systems, however, functional dispersion in the Dry Chaco was higher in open pastures compared to native forest. According to our results, native forests and forests with cattle maintain functional diversity in all regions. However, in the case of open pastures, the response depends on the regional context; the replacement of native forest by open pastures strongly affected functional diversity in humid forests and showed less impact on dry forest.

## Introduction

Livestock is one of the main land uses worldwide, covering more than 25% of the global surface^1,2^. In the last decades, livestock was one of the principal causes of forest replacement in tropical and subtropical areas^3,4^. The replacement of native forests by cattle pastures strongly modifies biotic and abiotic conditions, resulting from the loss of tree cover and soil changes^5^. These changes modify the conditions of environmental filters which in turn affect the abundance, richness, and composition of biological communities and populations^6^. While livestock activities, mainly by grazing and trampling, in general, negatively affects native communities, the response of species to canopy loss is variable; native or exotic species adapted to open habitats are often positively affected while species requiring denser cover are negatively affected^7^.

Environmental filtering has been proposed as an assembly-forming mechanism at different scales^8^. Kraft et al.^9^ define environmental filtering sensu stricto, as environmental conditions and resources that prevent species with physiological or ecological requirements from establishing or persisting in a specific area. Environmental filters act on species traits, allowing the establishment and persistence of species with certain traits of specific conditions and excluding others^9^. Consequently, these ecological filters are generally structured hierarchically and select, or filter, a subset of species sharing functional traits from the regional pool, conforming local communities^8,10,11^. In the particular case of human disturbed-habitats, this mechanism has been proposed as the main driver in the formation of new species assemblages after disturbance^12^.

Climate acts as a regional filter that guides the distribution of species according to their tolerance to environmental conditions and resources requirements^13^. In particular, temperature and precipitations have a strong influence on both diversity and abundance patterns^14^. The role of climate determining functional patterns of diversity at the regional scale has been explored for several taxa, including plants^15^, insects^16^, and mammals^17^; among others. Previous studies in tropical and subtropical areas showed that the rainfall regime is usually the strongest predictor of regional functional diversity patterns at a regional scale^18,19^. In contrast, at a smaller scale, anthropogenic disturbance imposes local filters modifying the functional structure of local communities beyond changes in species richness^20,21^.

Dung beetles (Scarabaeidae: Scarabaeinae) are one of the most diverse taxa and a focal group in ecological studies^22^. Through organic matter burial, dung beetles have a central role in ecosystem functioning^22,23^. Considering the central functional role of these taxa, functional diversity provides a useful theoretical background to understand the effects of human land use on ecosystem processes mediated by species. Functional diversity can be comprehensively described using three functional indices. Functional richness (Fric) represents the volume of the functional space occupied by a community^24,25^. The functional evenness (Feve) measures the uniformity of the distribution of species abundances in the functional space^24^. Finally, the functional dispersion (Fdis) represents how far species are from the center of the functional space^26^. Several recent studies have evaluated the effect of livestock on the functional diversity of dung beetles using functional indices^27–30^. From these studies, two general patterns emerged. On one side, in humid subtropical and tropical forests with low or no seasonality, functional diversity is strongly and negatively affected by livestock. On the other side, in dry and xerophytic seasonal forests, livestock has lower, or even positive, effects on dung beetle functional diversity.

While previous studies focused on specific ecosystems, for the first time we simultaneously compared the functional response of dung beetles to livestock grazing, in forests with contrasting climatic conditions in the subtropic of Argentina. We also explored the role of local and regional factors influencing local and regional responses to this land uses.

## Methods

### Study area and experimental design

In this manuscript, we used primary data from a previous study^31^ and we measured a large database on morphometric and ecological traits for the species considered in this study. Sampling was performed between 2015 and 2017 in the spring season, the time of year with the highest activity of dung beetles in Neotropical forests^32^.

Three subtropical forest regions were sampled: the Atlantic Forest (AF), the Humid Chaco (HCh), and the Dry Chaco (DCh). According to Köppen's classification, the climate for the Atlantic Forest and Humid Chaco regions is Cfa (warm temperate, fully humid, and hot summers). In the Dry Chaco, the climate is Cwa (warm temperate with dry winter, and hot summer)^33^. The three regions present similar temperature ranges (20 °C for AF, 22 °C for HCh and 23 °C for DCh) and differ, mainly, in the total amount and seasonal pattern of precipitation: 1600–2000 mm with low seasonality in the Atlantic Forest, 750–1300 mm with medium seasonality in the Humid Chaco and 500–700 mm with high seasonality in the Dry Chaco^33,34^. In both Humid and Dry Chaco, precipitations are concentrated in spring–summer.

Within each region, we selected five replicates of three habitat types: open pastures with cattle, native forests with cattle, and native forests in protected areas without cattle (for a detailed description see Appendix S1 of Supporting Information). In the habitat type “forest with cattle”, farmers directly introduce cattle inside the forest, without any specific silvicultural management; animals opportunistically feed on native understory vegetation. The habitat type “open pastures” refers to deforested areas with implanted exotic pastures. Replicates within each sampling area were separated by at least 1000 m to reduce their dependence.

### Dung beetles collection

For the dung beetle collection, 10 pitfall traps separated by 50 m were installed on each sampling site (three regions × 2 years × three habitats × five replicates × 10 traps = 900 traps). Traps were baited intercalated with human feces and rotting meat to attract coprophagous and necrophagous beetle species^23,35^. In each area, the sampling time was eight days, and the bait was renovated every 48 h. Species were determined through consultation with specialists and the use of taxonomic keys^36^. For more details on the collection of dung beetles see^31^.

### Local and regional vegetation structure and environmental conditions

To describe vegetation structure at the local scale, we established three sub-plots of 5 × 15 m on each replicate of each habitat type and region (three plots × three regions × three habitats × five replicates = 135 plots). The three subplots within each replicate were averaged. In each subplot, four variables were estimated based on an abundance Braun-Blanquet's cover scale^37^ (0–100%) (1979): (1) bare soil, (2) herbaceous vegetation, (3) shrub vegetation, and (4) canopy cover. Automatic sensors (HOBO ProV2) were used to estimate temperature and humidity on each sampling site during field sampling. Automatic sensors recorded both variables every five minutes. From sensors data, the average maximum and minimum daily temperature were calculated. Additionally, the thermal amplitude was calculated by subtracting both minimum and maximum daily temperature. For more details on the local environmental characterization see^31^.

At the regional scale, three bioclimatic variables were selected and calculated for each sampling site: (a) mean annual temperature (BIO1), (b) mean day range (mean monthly temperature (maximum temperature − minimum temperature)) (BIO2), and (c) seasonality of precipitation (coefficient of variation) (BIO15). These variables were extracted from the WorldClim database^38^ and represent an average for the period 1970–2000 with a spatial resolution of 30 s (~ 1 km^2^)^38^.

### Characterization of functional traits

**Figure 1:**
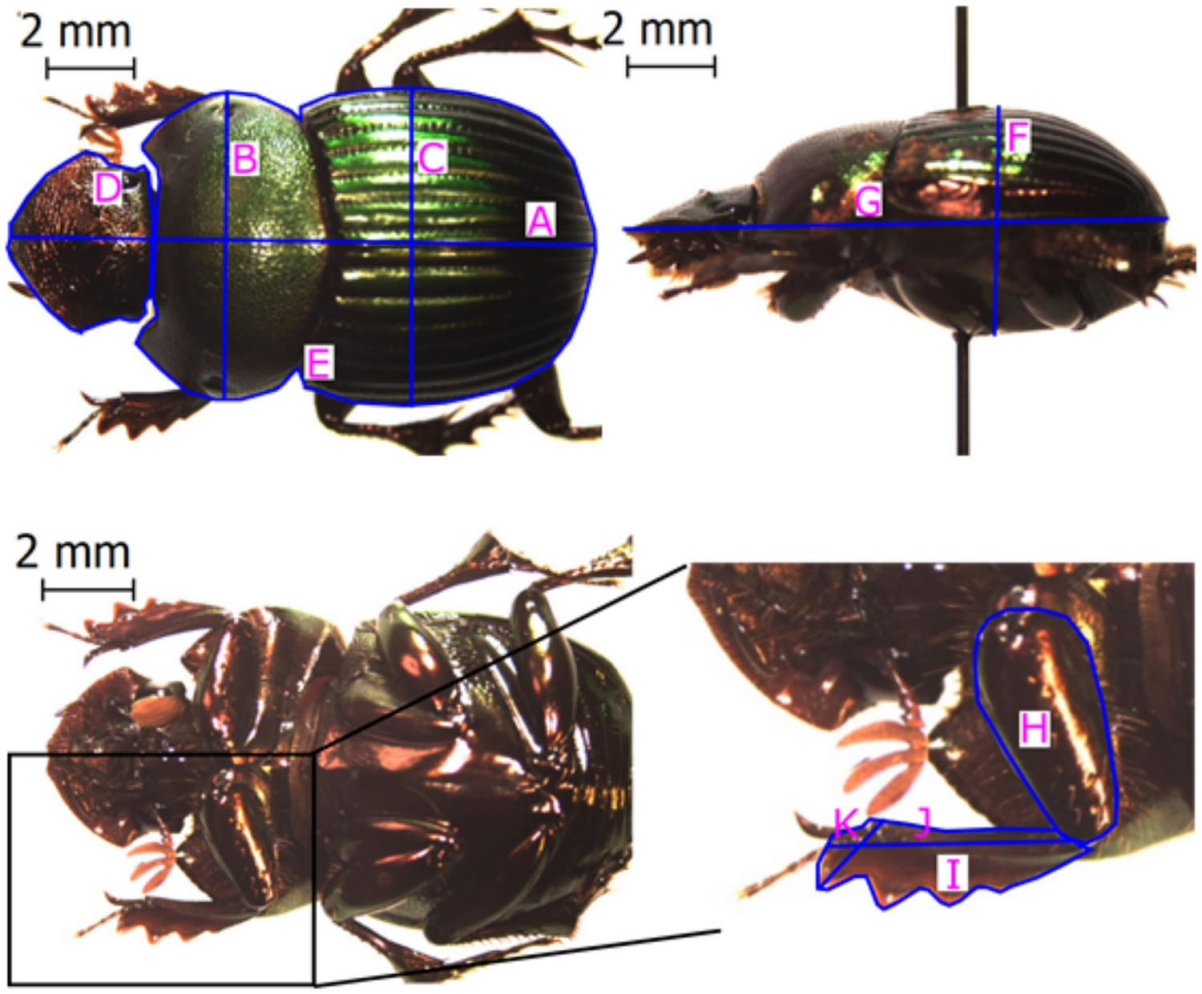
Dorsal, lateral, and ventral view of Chalcocopris hesperus. A: Total length, B: Pronotum width, C: Elytra width, D: Head area, E: Body area, F: Depth, G: Lateral length, H: Femur area, I: Anterior tibia area, J: Anterior tibia length, K: Width of the largest tooth in the anterior tibia.

Considering the sexual dimorphism of this taxon, three to 10 females of each species were selected for morphometric description. Individuals were independently selected in each region; in the case of species occurring in more than one region, individuals of each region were measured independently. In total, 448 females from 71 species were measured; 25 species and 131individuals in the Atlantic Forest, 213 individuals of 38 species for the Humid Chaco and for the Dry Chaco, 104 individuals of 23 species (see the data abailability for a complete list of traits). Each individual was photographed in dorsal, ventral, and lateral views (Fig. 1) using a LEICA EZ4 D magnifier and LASEZ software (Leica Application Suite) (version 3.3.0, https://www.leica-microsystems.com/).

Once the images were taken, imageJ software (version 1.49, https://imagej.nih.gov/ij) was used to estimate 10 morphological response traits related to the ecology and physiology of dung beetles: (1) body area, (2) biomass, (3) total length, (4) sphericity, (5) head area, (6) pronotum width, (7) anterior tibia length, (8) anterior tibia area, (9) anterior femur area and (10) tooth width (Fig. 1 and Appendix S2 Supporting Information). Sphericity was calculated using the formula: (S2/LI) 1/3, where L is the total length of the body, I is the width of the elytra, and S is the thickness of the body (Table 1). Biomass was measured using an analytical digital balance with a 0.0001 g precision. The ecological roles that are represented in the traits are shown in Table 1. Additionality, we included the relocation food pattern as a qualitative trait with three categories: telecoprid, paracoprid and endocoprid. This trait is associated with the capacity of manipulation and the amount of organic matter buried by dung beetles and was defined from previous studies^39–42^. In total, we used 11 traits.

**Table 1:** Functional traits of dung beetles and their ecological role.

Functional trait	Morphological measurement	Ecological role
Body size	Body area	Distribution of organic matter and amount of resources consumed in the larval stage^1–3^
	Biomass	
	Total length	
Body shape	Sphericity	Distribution of organic matter and amount of resources consumed in the larval stage. Substrate penetration capacity (soil and food resources)^1,3,4^
Head size	Head area	Substrate penetration capacity (soil and food resources)^2,4^
Muscular mass	Pronotum width	Substrate penetration capacity (soil and food resources)^2,4–6^
	Anterior tibia length	Ability to dig, necessary for food handling and nesting^2,4,7–10^
	Anterior tibia area	
	Anterior femur area	
Tibial tooth size	Tooth width	Ability to dig, to escape from predators and to reproduce^2,11^

The references of this table are in Appendix S2 in Supporting information.

### Statistical analyses

First, a simple regression analysis was performed between each trait and the body area to explore the allometric relationships for species in each region. The allometric analysis was based on the conventional allometric equation Y = aX^b^, where Y is any morphological trait (dependent variable), X is the body area (independent variable), and a and b are the allometric coefficients^43^. For those traits that showed significant allometric relationships, residuals of the simple regression models were extracted to create new morphological traits independent of the body area. These corrected variables (regression residuals) were then used for single and multiple trait comparisons^44^.

Morphological traits in all regions, except the sphericity, were positively and strongly related to the body area (allometric dependence) (Appendix S6—Supporting Information). Therefore, for all morphological variables (except sphericity) the residuals of the allometric relationships were used as independent variables in statistical analysis.

Three indices were used to explore the amount of the functional space occupied by species and its distribution on each region and habitat type, using the ‘FD’ package^45^. Functional richness (Friq) considers all traits and their values within the functional space, without considering the relative abundance^25^. Functional evenness (Feve) measures the level of regularity of the functional assemblage; this index distributes abundances within the functional space^25^. Functional dispersion (Fdis) is the average distance of all species from the centroid of the functional space taking into account relative abundances^26^; Fdis describes the degree of heterogeneity of functional traits within an assemblage and increases with the number of ecological processes. To estimated functional indices, relocation food was considered a single variable with three levels: telecoprid, paracoprid and endocoprid. For the calculation of the indices, the species distance matrix based on each trait was constructed using the ldist.ktab and dist.ktab functions of the ‘ade4’ package. These functions are based on Gower's^46^ general distance measure and allow dealing with qualitative and quantitative traits^47^.

After functional indices estimation, generalized linear mixed models (GLMM) were performed to explore the effects of region and habitat types (explanatory variables) on functional diversity (Friq, Feve, and Fdis). To evaluate the assumption of spatial independence of observations, the Moran's index I^48^ (Moran, 1950) was calculated for the residuals of GLMM analysis as an overall measure of spatial autocorrelation; using SAM (version v4.0, https://ecoevol.ufg.br/sam/). Since no autocorrelation of the residuals was observed in any of the three models (Friq, Feve, and Fdis), geographical coordinates of sampling sites were not included in GLMM analysis (Appendix S3—Supporting Information).

To explore the role of local and regional environmental variables to explain patterns of functional diversity, three independent principal component analyses (PCAs) were first conducted to reduce the number of explanatory variables. Then, the first axis of each PCA was used as fixed factors in the GLMMs as explanatory variables. The first PCA was performed with local vegetation structure: canopy cover, shrub cover, herb cover, and bare soil cover. The second with local microclimatic conditions: thermal amplitude, average daily temperature and humidity, and average daily maximum temperature. And the last PCA with regional climatic variables: average annual temperature, average daytime temperature range, seasonality of precipitation. Normality and homocedasticity were evaluated through residuals vs. predicted and qqnorm plots. Finally, each model was compared with its respective null model to determine the significance of the factors analyzed. Collinearity between the first axis of each PCA (predictor variables of the models) was evaluated through the vif function of the ‘car’ package^49^.

To describe changes in the functional structure of dung beetles assemblages, the per-site weighted mean of each trait (CWM)^50^ was calculated with the functcomp function of the ‘FD’ package^45^. Two matrices were constructed, one with the abundance of individuals of each species per site (replicate), and the other with the functional traits of each species. Based on these matrices, a new matrix was generated with the values of the traits per site. From this new matrix, the variation explained by space (spatial coordinates) and environmental variables was estimated through variation partition analysis with the varpart function of the ‘vegan’ package^51^. To calculate the cwm, relocation food was considered three different variables (proportion of telecoprids, endocoprids, and paracoprids).

The partitioning of the variation showed that the spatial structure of the data had only a small influence on the variation in trait structure among habitat types and regions (7%) (see Appendix S4—Supporting Information); consequently, the spatial location of the sites was excluded as an explanatory variable in further analysis of the functional structure. An RDA analysis was then carried out to evaluate if the ordering of sites according to functional traits from CWMs was associated with environmental variables. This analysis was performed through an ANOVA based on permutations (9999 permutations restricted inside each region) with the ‘vegan’ package^51^. This analysis used a stepwise variable selection procedure to determine predictors with the highest proportion of explained variation in all models. Prior to the analysis, environmental variables were standardized and transformed with square root to reduce the influence of extreme outliers^52^. In case of multicollinearity among environmental variables (> 0.6), one of each pair was selected and the other was excluded from the analyses (Appendix S5—Supporting information). Finally, the effect of region, habitat types and the interaction between factors on groups formed was evaluated through a multivariate permutational analysis of variance (PERMANOVA), using the adonis function of the ‘vegan’ package^51^. Finally, to evaluate how traits vary by region and habitat type, generalized linear mixed models were performed for each trait. Only three traits fit the GLMM assumptions (normality of residuals and homogeneity of variances) the pronotum width, head area and femur area. All statistical analyses were performed in the R software (https://www.R-project.org/).

## Results

**Figure 2:**
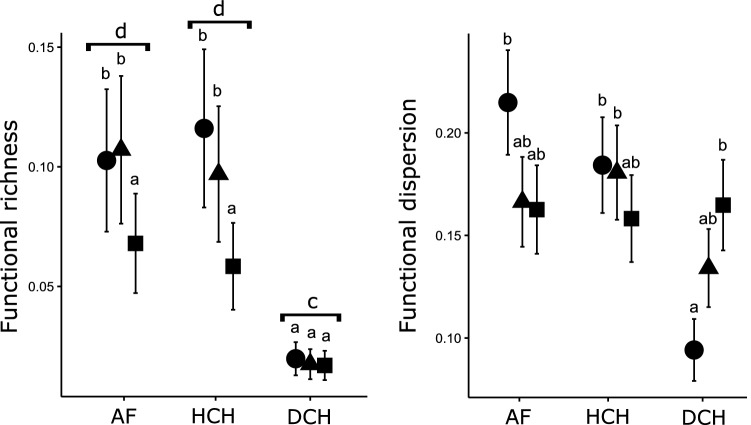
Dung beetle functional richness and dispersion among different regions and habitat types in subtropical forests of Argentina based on a GLMM analysis. AF: Atlantic Forest, HCH: Humid Chaco, DCH: Dry Chaco. Circles: native forest, triangles: forests with cattle, squares: open pastures. Different letters indicate significant differences (*p* < 0.05). Brackets indicate differences in functional richness among regions.

Based on the GLMM analysis, functional richness was higher in native forests and forest with cattle when compared to open pastures (Fig. 2, Table 2). For the regions, the Atlantic Forest and the Humid Chaco showed higher and similar functional richness compared with the Dry Chaco. The functional richness did not show a significant interaction between factors (region × habitat types). Functional evenness (Feve) was similar between regions and habitat types (Table 2) whereas functional dispersion (Fdis) in the Atlantic Forest and the Humid Chaco was similar between native forest and forest with cattle and open pasture. Finally, in the Dry Chaco, Fdis was higher in pastures compared to the native forest whereas forests with cattle represent an intermediate situation (Fig. 2, Table 2).

**Table 2:** GLMM analysis considering the region, the habitat types, and their interaction on dung beetles functional richness (Fric), functional evenness (Feve), and functional dispersion (Fdis) in subtropical forests of Argentina.

Index	Factors	*d.f.*	χ^2^	*p*
Fric		8	40.89	< 0.0001
	Region	2	17.66	< 0.0001
	Habitat type	2	40.13	< 0.0001
	Region × Habitat type	4	7.51	0.111
Feve		8	9.927	0.270
	Region	2	0.47	0.791
	Habitat type	2	4.35	0.114
	Region × Habitat type	4	5.47	0.242
Fdis		8	23.4	0.002
	Region	2	4.73	0.094
	Habitat type	2	0.27	0.873
	Region × Habitat type	4	21.43	0.0002

*d.f.*: degrees of freedom.

The first axis of the three PCAs performed to reduce the number of explanatory variables (local vegetation structure, local microclimate conditions and regional climate) explained more than 50% of the variation in all cases (Appendix S7—Supporting Information). The GLMM analysis using this first axis of these PCA, showed that the functional richness was mainly explained by local vegetation structure (χ^2^ = 6.23, *P* < 0.012), while local microclimate conditions and regional climate had no effect (χ^2^ = 0.92 and 0.45, respectively, *P* > 0.1 in both cases). In the case of functional evenness none of the environmental variables explained observed patterns (Vegetation: χ^2^ = 0.09, *P* = 0.76; Microclimatic conditions: χ^2^ = 2.68, *P* = 0.10; Regional climate: χ^2^ = 1.22, *P* = 0.26) same as for Fdis (Vegetation: χ^2^ = 0.93, *P* = 0.33; Microclimatic conditions: χ^2^ = 1.61, *P* = 0.20; Regional climate: χ^2^ = 1.37, *P* = 0.24).

**Figure 3:**
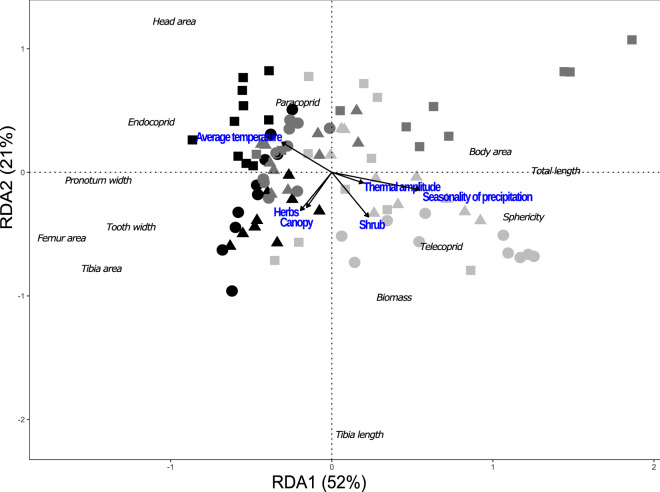
Redundancy analysis based on functional traits of dung beetle assemblages in the native forest and two cattle systems in subtropical forests of Argentina. Black symbols: Atlantic Forest, dark grey symbols: Humid Chaco, light gray symbols: Dry Chaco. Circles: native forest, Triangles: forest with cattle, and Squares: open pasture.

The first axis of the redundancy analysis (RDA) explained 52% of the changes in functional structure (composition and abundance) of dung beetles among regions and habitat types. In this axis, native forest and forests with cattle of the Atlantic Forest and the Humid Chaco formed a single group and separated from the Dry Chaco. The second axis explained 21% of the variation and separated the Atlantic Forest and Humid Chaco pastures from the rest of the sites (Fig. 3). PERMANOVA analysis validated these groups (F Model = 10.84, R^2^ = 0.52, g. l. = 8, *P* = 0.0001); according to the region (F = 19.03, R^2^ = 0.23, g.l. = 2, *P* = 0.0001), the habitat types (F = 4.52, R^2^ = 0.05, g.l. = 2, *P* = 0.0008) and the interaction of both factors (F = 9.90, R^2^ = 0.24, g.l. = 4, *P* = 0.0001).

Environmental variables explained 36.32% of changes in the functional structure of RDA formed groups (g.l. = 6, F = 7.88, *P* = 0.0004). The first axis was negatively associated with local mean temperature, canopy, and shrub cover and was positively associated with seasonality of rainfall, thermal amplitude, and shrub cover. The second axis was positively correlated with mean temperature and negatively with the other variables (Table 3). Native forest and forests with cattle in the Atlantic Forest and Humid Chaco were mainly characterized by a higher canopy and herb cover. In contrast, the Atlantic Forest open pastures were characterized mainly by higher local mean temperature. Finally, sites in the Dry Chaco were associated with higher seasonality of rainfall, thermal amplitude, and shrub cover (Fig. 3).

**Table 3:** Role of local (L) and regional (R) environmental variables in an RDA analysis, explaining the functional structure of dung beetle assemblages in native forest and two livestock systems (forest with cattle and open pasture) in subtropical forests of Argentina (Atlantic Forest, Humid and Dry Chaco).

	F	*p*
Seasonality of precipitation (R)	10.96	0.07
Canopy cover (L)	9.58	0.0001
Shrub cover (L)	7.48	0.0006
Average temperature (L)	3.37	0.09
Thermal amplitude (L)	9.67	0.0001
Herbaceous cover (L)	3.77	0.005

Degrees of freedom: 1.

**Figure 4:**
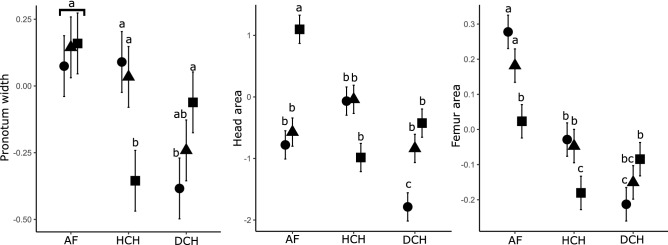
Pronotum width, head area and femur area of dung beetle among different regions and habitat types in subtropical forests of Argentina based on a GLMM analysis. AF: Atlantic Forest, HCH: Humid Chaco, DCH: Dry Chaco. Circles: native forest, triangles: forests with cattle, squares: open pastures. Different letters indicate significant differences (*p* < 0.05).

In relation to individual traits, native forest and forest with cattle of the Atlantic Forest and the Humid Chaco were characterized by species with larger head area, pronotum width, tibia, and anterior femur area, and by a higher frequency of endocoprid and paracoprid species (Fig. 3). In contrast, Dry Chaco and the pastures in the Humid Chaco were characterized by species with larger body area, total length, biomass, more spheric, and higher frequency of telecoprids (Fig. 3). Regarding the GLMMs of traits, it is observed that the pronotum width is greater for the Atlantic Forest, and does not differ among habitat types, in the Humid Chaco it is greater in native forests and forests with cattle and smaller in open pasture. In the Dry Chaco pronotum width is lower in native forests and higher in open pastures, being intermediate in forests with cattle. As for the head area in the Atlantic Forest, it is higher in pastures and lower in native forests and forests with cattle. In the Humid Chaco it does not differ among habitat types. In the Dry Chaco the head area is greater in open pastures and forests with cattle and smaller in native forests. As for the femur area in the Atlantic Forest and in the Humid Chaco, it is greater in native forests and forests with cattle and smaller in open pastures. In the Dry Chaco, the femur area is inverted, being smaller in native forests, larger in open pastures and intermediate in forests with cattle (Fig. 4, Table 4).

**Table 4:** GLMM analysis considering the region, the habitat, and their interaction on pronotum width, head area and femur area of dung beetles.

Traits		*d.f.*	F	*p*
Pronotum width	Region	2	3.008	0.191
	Habitat type	2	0.976	0.381
	Region × habitat type	4	11.448	2.32E − 07
Head area	Region	2	8.496	0.058
	Habitat type	2	11.613	3.85E − 05
	Region × habitat type	4	17.901	1.83E − 10
Femur area	Region	2	15.537	0.026
	Habitat type	2	9.0407	0.0002
	Region × habitat type	4	12.575	6.07E − 08

*d.f.*: degrees of freedom.

## Discussion

While previous studies explored either local or regional functional responses of dung beetles to environmental gradients or human disturbances^29,30,53–55^; this is the first study that simultaneously explore both components through the comparison of similar land uses among different regions. Consistent with previous studies, the replacement of native forest by open pastures strongly reduced functional diversity in humid forest and showed low impact on dry forests^27,29,56^. In all regions, forests with cattle preserving native canopy also preserve the same functional diversity as native forest ^28,57^.

Seasonality and the total amount of precipitation were the major differences among regions in this study. Consistent with this pattern, Nichols et al.^55^, proposed that the functional response of dung beetles to anthropogenic disturbance depends on the regional context whereas da Silva and Cassenote^53^ proposed that climatic seasonality is the main determinant of functional dung beetles response. Environmental dissimilarity between native habitats and land uses has already been identified as one of the main predictors of changes in biological assemblages at both local and regional scales^20,58^. The extreme temperatures and simplified vegetation in the open pasture and the low tolerance of native humid forest dung beetles to these conditions may influence the loss of functional (and taxonomic) diversity in both the Atlantic Forest and the Humid Chaco^59–63^. In contrast to humid forests, in the Dry Chaco, native forests and pastures showed less contrast on environmental conditions and native dung beetles are probably more tolerant to extreme conditions. To sum up, differences in environmental similarity among regions and land uses, and the tolerance of native species to extreme conditions could explain the differences in the functional response of the assemblages between the humid forests (Humid Chaco and Atlantic Forest) and the Dry Chaco.

Similar to our results on forests with cattle, Cerullo et al.^57^ found that functional richness and dispersion (Fric and Fdis) of Borneo dung beetles in silvopastoral systems were similar to the ones observed in undisturbed forests. These systems, in contrast to open pastures, partially maintain microclimatic and soil conditions, which in turn, would favor the persistence of native species. In addition, other factors regulated by the canopy cover (such as light intensity and heat) may influence the observed patterns of functional diversity of dung beetles between open and closed habitats^64,65^. Several previous studies have shown the importance of vegetative cover to preserve dung beetle assemblages^66–68^.

The higher functional dispersion in pastures, compared to the native forest, of the dry Chaco suggests that anthropogenic disturbance generates a greater heterogeneity in the distribution of functional trait status; these results coincide with other studies conducted in xerophytic forests in Mexico^28^ as well as in the Cerrado in Brazil^29^. As in these previous studies, no evidence was found that cattle ranching reduces the functional richness of dung beetle communities in arid environments. One possible explanation is that the dry Chaco is a semi-arid region, inhabited by species that can use open areas lacking vegetation and can take advantage of the additional manure provided by livestock^28,69^. Fric and Feve's neutral response to cattle ranching in the dry Chaco could result from the balance between individuals and species loss and gains from adjacent habitats, which result in assemblages of similar functional trait space (Fric), or in the regularity of trait abundance distribution (Feve), regardless of species identity.

In turn, Davis et al.^70^ concluded that certain functional traits, such as the ability to roll balls, predominate in arid regions. Consistent with this pattern, rolling ball species (telecoprid) predominate in the Dry Chaco whereas in humid forests paracoprid and endocoprid predominate. The loss of canopy and the high thermal amplitude given in open pastures increase the drying rate of dung in open pastures and (particularly in open pastures of the Dry Chaco). Since dung beetles depend on the liquid component of dung for feeding, the water loss in dung paths reduces food quality and may explain the low abundance of endocoprid species in this habitat (dung beetles inhabiting inside dung paths). The absence of paracoprid species in pastures could be associated with the higher temperature and compaction as well as lower soil moisture in these habitats, leading to rapid drying of the manure and obstructing the handling and burying of the resource in the soil. On the other hand, telecoprid beetles have the ability to transfer the food to a more suitable place for burial and may therefore be able to persist in open habitats^71^.

Regarding the observed traits, for the most humid regions (Atlantic Forest and Humid Chaco), species with large femurs were typical forests (with and without cattle), which is probably associated with the ability of certain species to bury organic matter and the paracoprid's ability to tunnel. On the other hand, and contrary to what we expected and to previous studies^72^, the presence of large species with bodies, pronotum, heads and femurs larger in open pastures of the Dry Chaco can be associated to the thermoregulatory capacity of certain species, or the nocturnal or crepuscular activity which would enable them to make use of open habitats^73,74^. These morphological characteristics allow them a greater burial capacity in arid soils exposed to extreme temperatures and with little vegetation, which generates a decrease in water content in soils and consequently greater hardness^75^.

The opportunistic and extensive introduction of cattle inside the forest is an extended practice in humid and dry tropical and subtropical forests (namely forest with cattle in our study)^56,76,77^. Whereas this practice usually results in a degradation of habitat quality for animals^78,79^, functional dung beetle assemblages were not particularly affected by this livestock grazing. As we previously mentioned, the maintenance of canopy cover (particularly in humid forests) is the key management tool to maintain dung beetles diversity and their functional roles.

According to our results, as well as with a growing amount of recent evidence, the functional response of dung beetle assemblages to livestock grazing cannot be generalized for all biomes. Regional climate, the interaction between scales (local and regional), and the environmental similarity between native habitats and land uses are the major determinants of this response. In addition to taxonomic diversity, the preservation of functional diversity is of central importance to maintaining ecosystem functioning^80,81^. This is particularly important in livestock areas considering the central role of dung beetles in manure processing^82^. The results of this study provide a basis for future questions regarding the knowledge of how functional processes may be affected by changes in functional diversity in subtropical forests and how these changes may vary depending on the region and livestock grazing. In turn, these results contribute to the knowledge needed for decision-making in terms of biodiversity conservation in subtropical forests affected by land uses, in particular livestock grazing.

## Supplementary Information

Supplementary Information.

## Data Availability

The datasets generated and/or analyzed during the current study are available in the Figshare repository. https://doi.org/10.6084/m9.figshare.13313186.v4.
