# Supplementary material for: Livestock grazing impact differently on the functional diversity of dung beetles depending on the regional context in subtropical forests
Source: Sci Rep. 2022 Jan 31;12:1636. doi: 10.1038/s41598-022-05616-x (PMC8803855; doi:10.1038/s41598-022-05616-x)
Supplement: Supplementary file 1 — Supplementary Information. [file 41598_2022_5616_MOESM1_ESM.pdf]

Livestock grazing impact differently on the functional diversity of dung beetles depending on the regional context in subtropical forests

Guerra Alonso Celeste B.[1]*; Gustavo A. Zurita[2]; M. Isabel Bellocq[34]

[1]Instituto de Biología Subtropical, Universidad Nacional de Misiones-CONICET Puerto Iguazú, Misiones, Argentina. https://orcid.org/0000-0003-0778-6969

[2]Facultad de Ciencias Forestales, Universidad Nacional de Misiones-CONICET, Eldorado, Misiones, Argentina.

[3]Departamento de Ecología, Genética y Evolución, Facultad de Ciencias Exactas y Naturales, Universidad de Buenos Aires, Buenos Aires, Argentina. [4]M. Isabel Bellocq is deceased.

*Email: clstguerra@gmail.com

Supporting Information

Appendix S1: Location of sampling sites

In the Atlantic Forest, sampling was performed in the north-central part of the province of Misiones (between 25°58'2" S, 54°17′22" W, and 26°36′32" S, 54°41′43" W) during the spring of 2015 and 2016 in the Iguazu National Park, Urugua-i Provincial Park, the San Jorge Private Reserve, and surrounding livestock establishments.

In the Humid Chaco, samples were conducted in two areas separated by 150 km: the northern area, located in the province of Formosa (between 25°59'24.2 "S, 58°10'48" W and 25°57'33.6 "S, 58°11'27. 1" W) and the southern zone located in the province of Chaco in the Chaco National Park and its surroundings (between 26°46'52.7 "S, 59°37'2.8" W and 26°50'6.6 "S, 59°36'17.6" W). The northern zone was sampled during the spring of 2015 and the southern zone was sampled during the spring of 2016.

In the Dry Chaco, sampling was conducted in Copo National Park and in livestock establishments near the park (25°44'52.8 "S, 61°43'33.6" W and 26°11'11.4 "S, 61°42'19.6" W). Sampling was conducted during the spring of 2016 and 2017 in both sites.

Appendix S2: **References of Table 1.**

1. Andresen, E. Effect of forest fragmentation on dung beetle communities and functional consequences for plant regeneration. *Ecography* **26**, 87–97 (2003).
2. deCastro-Arrazola, I., Hortal, J., Noriega, J. A. & Sánchez-Piñero, F. Assessing the functional relationship between dung beetle traits and dung removal, burial, and seedling emergence. *Ecology* **n/a**, e03138 (2020).
3. Emlen, D. J., Marangelo, J., Ball, B. & Cunningham, C. W. Diversity in the Weapons of Sexual Selection: Horn Evolution in the Beetle Genus Onthophagus (coleoptera: Scarabaeidae). *Evolution* **59**, 1060–1084 (2005).
4. Evans, M. E. G. & Forsythe, T. G. Feeding mechanisms, and their variation in form, of some adult ground-beetles (Coleoptera: Caraboidea). *Journal of Zoology* **206**, 113–143 (1985).
5. Pringle, J. W. S. The Motor Mechanism of the Insect Leg. *Journal of Experimental Biology* **16**, 220–231 (1939).
6. Vilhelmsen, L., Mikó, I. & Krogmann, L. Beyond the wasp-waist: structural diversity and phylogenetic significance of the mesosoma in apocritan wasps (Insecta: Hymenoptera). *Zoological Journal of the Linnean Society* **159**, 22–194 (2010).
7. Davis, A. L. V. Community organization of dung beetles (Coleoptera: Scarabaeidae): differences in body size and functional group structure between habitats. *African Journal of Ecology* **34**, 258–275 (1996).
8. Halffter, G. & Edmonds, W. D. *The nesting behavior of dung beetles (Scarabaeinae): an ecological and evolutive approach*. (Instituto de Ecología, 1982).
9. Hernández, M. I. M., Monteiro, L. R. & Favila, M. E. The role of body size and shape in understanding competitive interactions within a community of neotropical dung beetles. *Journal of Insect Science* **11**, 1–14 (2011).

10. Inward, D. J. G., Davies, R. G., Pergande, C., Denham, A. J. & Vogler, A. P. Local and regional ecological morphology of dung beetle assemblages across four biogeographic regions: Ecological morphology of dung beetle assemblages. *Journal of Biogeography* **38**, 1668–1682 (2011).
11. Linz, D. M., Hu, Y. & Moczek, A. P. The origins of novelty from within the confines of homology: the developmental evolution of the digging tibia of dung beetles. *Proceedings of the Royal Society B: Biological Sciences* **286**, 20182427 (2019).

Appendix S3: Autocorrelograms for Moran's spatial autocorrelation index for the residuals of the GLMM analysis of Fric, Feve, and Fdis. Significance was tested using 9999 permutations. The black circles indicate a non-significant correlation.

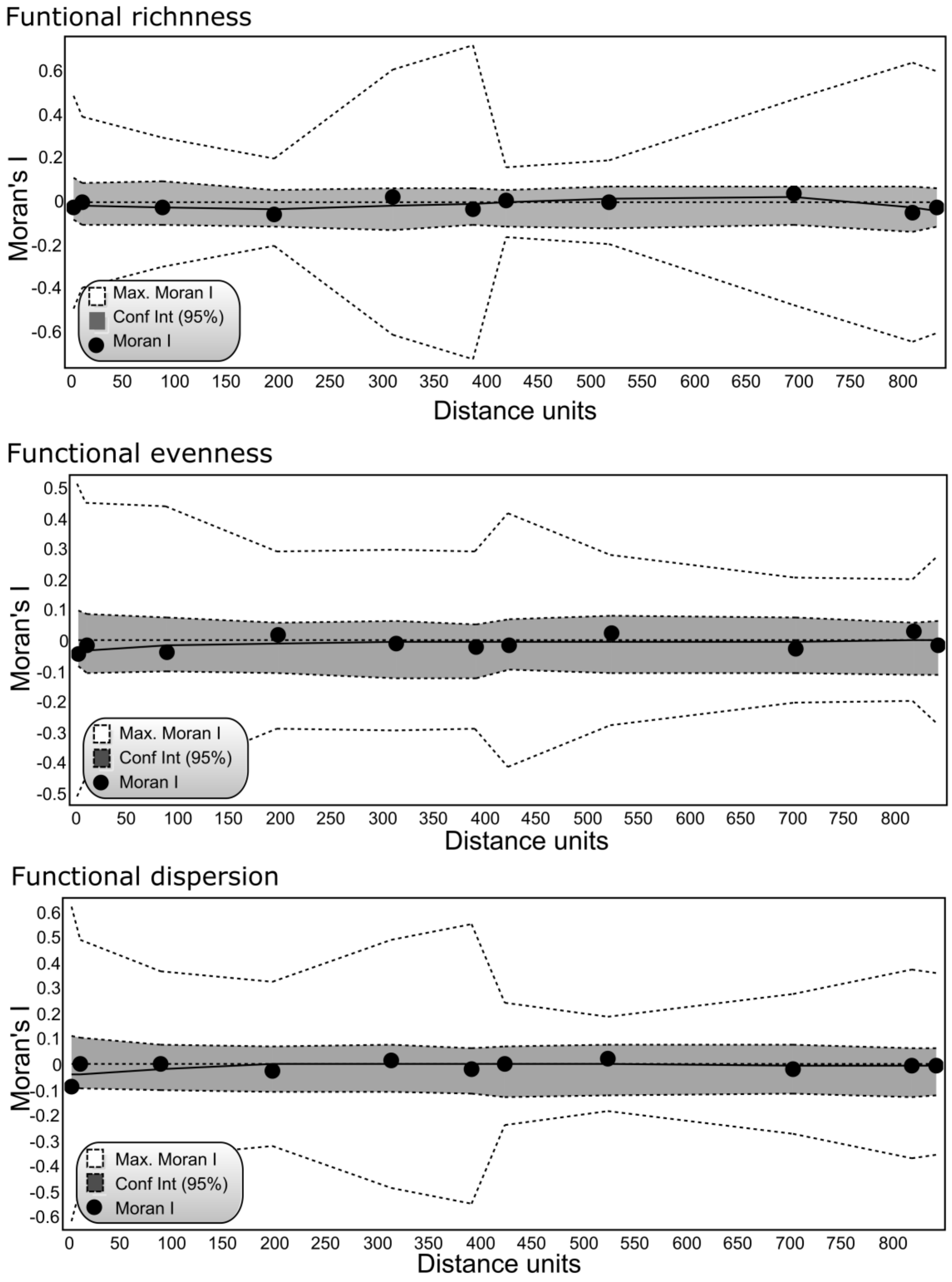

Appendix S4: Variation partitioning

According to the partitioning of variation, 32% (and 20% after control by spatial structure) of the observed variation in the functional structure of the dung beetle assemblages among land uses and regions was explained by all environmental variables (12), while spatial location (coordinates of the sampling sites) explained 19% (7% after control by environmental variables) (Figure 3A, Table 2A). The combined influence of all environmental variables and spatial location of the sites (geographic coordinates) explained 12% of the variation, whereas 61% of the variation was not explained by the model (residues).

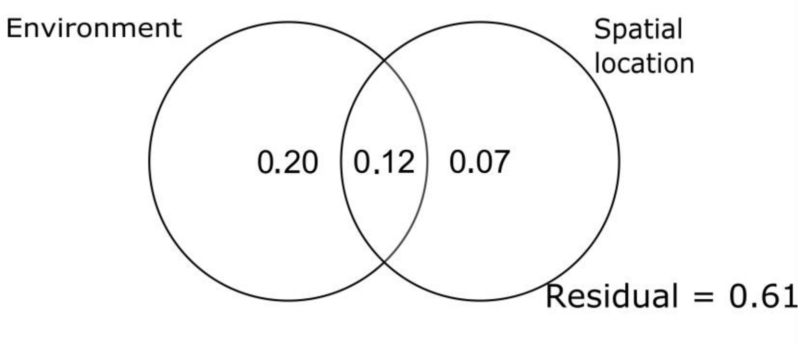

Figure S4.1: Variation partitioning analysis (proportion of variance explained) of environmental variables and spatial location explaining functional structure patterns (community weighted means - CWM) of dung beetle assemblages among the native forest, forest with cattle, and open pastures in three subtropical forests of Argentina (Atlantic Forest and Humid and Dry Chaco).

Table S4.1: Variation partition for the influence of spatial location (coordinates) and environmental variables on the functional structure (community weighted means - CWM) of dung beetles in three subtropical forests of Argentina (Atlantic Forest and Humid and Dry Chaco).

	**g.l.**	**R^2 Adj**	**F**	**P**
**Environment**	6	0.32	4.99	0.001
**Spatial location**	2	0.18	5.86	0.003

**Both**	8	0.38		
**Environment | Spatial location**	6	0.20	3.79	0.003
**Spatial location | Environment**	2	0.07	2.76	0.068
**Residues**		0.61		

Appendix S5: Correlation among environmental variables, measured in the native forest, forest with cattle, and open pastures within each region; the variables in blue font are those that were selected for the analyses, with a correlation below 0.6.

	**Average annual temperature**	**Mean Diurnal Range**	**Seasonality of precipitation**	**Thermal amplitude**	**Relative humidity**	**Average temperature**	**Maximum temperature**	**Bare soil**	**Canopy**	**Shrub**	**Herbaceous**
**Average annual temperature**	1.00	0.45	0.81	0.28	-0.38	-0.39	0.06	0.58	-0.12	0.10	-0.28
**Mean Diurnal Range**	0.45	1.00	0.83	0.69	-0.80	-0.46	0.38	0.65	-0.21	0.33	-0.10
**Seasonality of precipitation**	0.81	0.83	1.00	0.54	-0.66	-0.53	0.22	0.73	-0.13	0.29	-0.27
**Thermal amplitude**	0.28	0.69	0.54	1.00	-0.85	0.16	0.88	0.27	-0.60	-0.08	-0.26
**Relative humidity**	-0.38	-0.80	-0.66	-0.85	1.00	0.07	-0.66	-0.53	0.30	-0.26	0.19
**Average temperature**	-0.39	-0.46	-0.53	0.16	0.07	1.00	0.53	-0.47	-0.36	-0.44	-0.03
**Maximum temperature**	0.06	0.38	0.22	0.88	-0.66	0.53	1.00	0.04	-0.64	-0.24	-0.19
**Bare soil**	0.58	0.65	0.73	0.27	-0.53	-0.47	0.04	1.00	0.23	0.53	-0.03
**Canopy**	-0.12	-0.21	-0.13	-0.60	0.30	-0.36	-0.64	0.23	1.00	0.64	0.37
**Shrub**	0.10	0.33	0.29	-0.08	-0.26	-0.44	-0.24	0.53	0.64	1.00	0.21
**Herbs**	-0.28	-0.10	-0.27	-0.26	0.19	-0.03	-0.19	-0.03	0.37	0.21	1.00

Appendix S6: Allometric relationships of morphometric traits of dung beetles

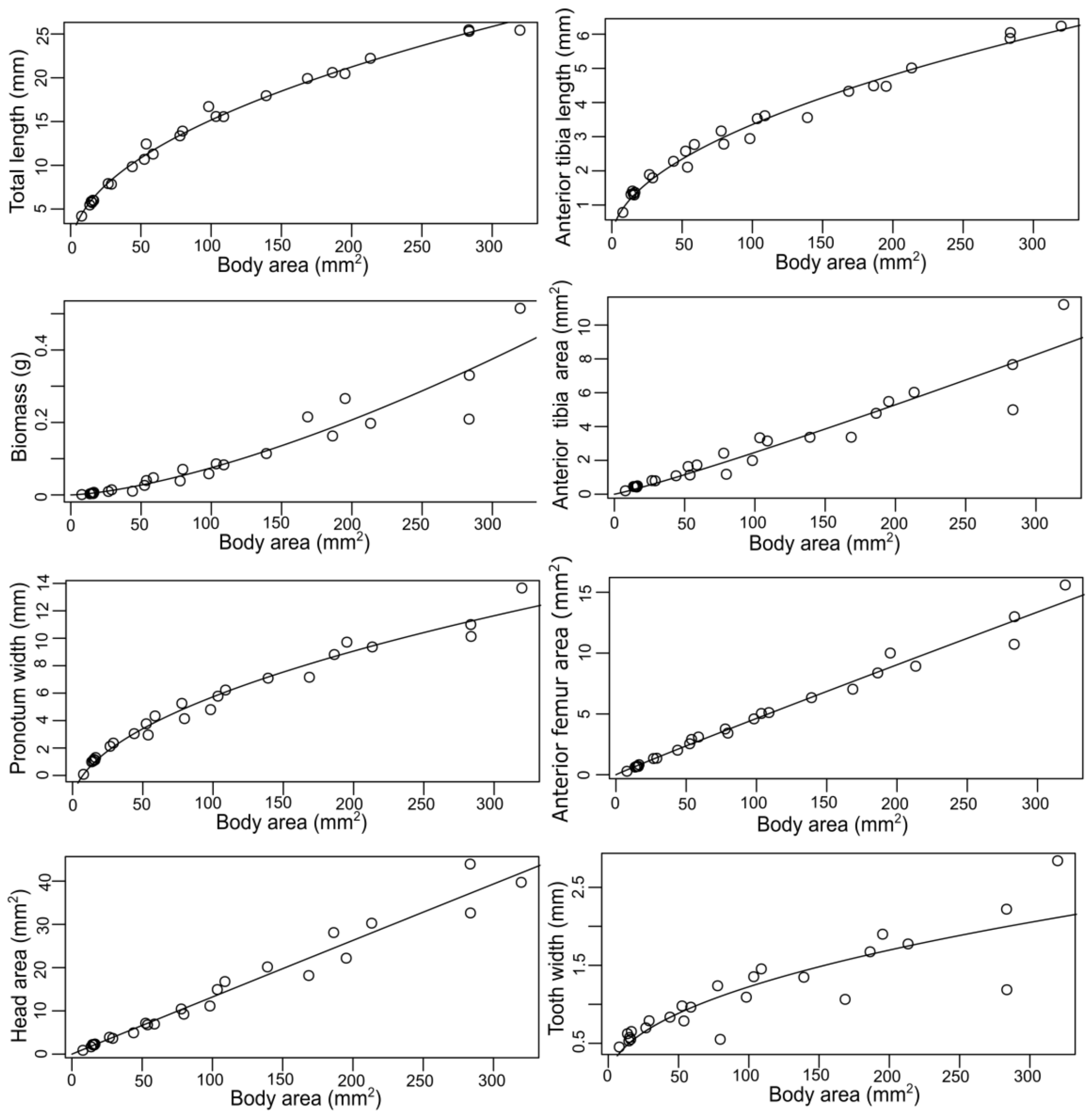

Figure S6.1: Allometric relationships of morphometric traits of female Atlantic Forest dung beetles.

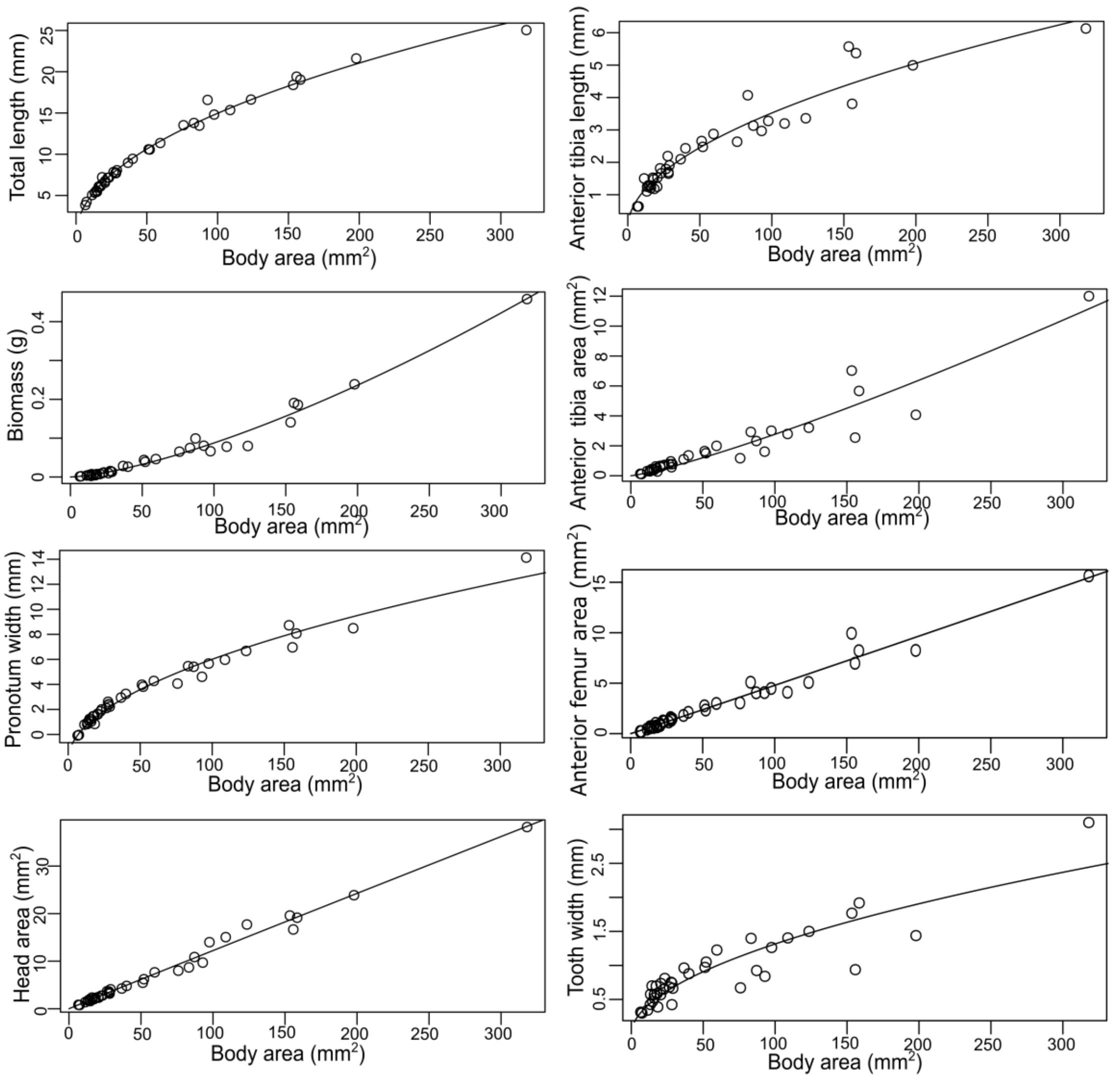

Figure S6.2: Allometric relationships of morphometric traits of female Humid Chaco dung beetles.

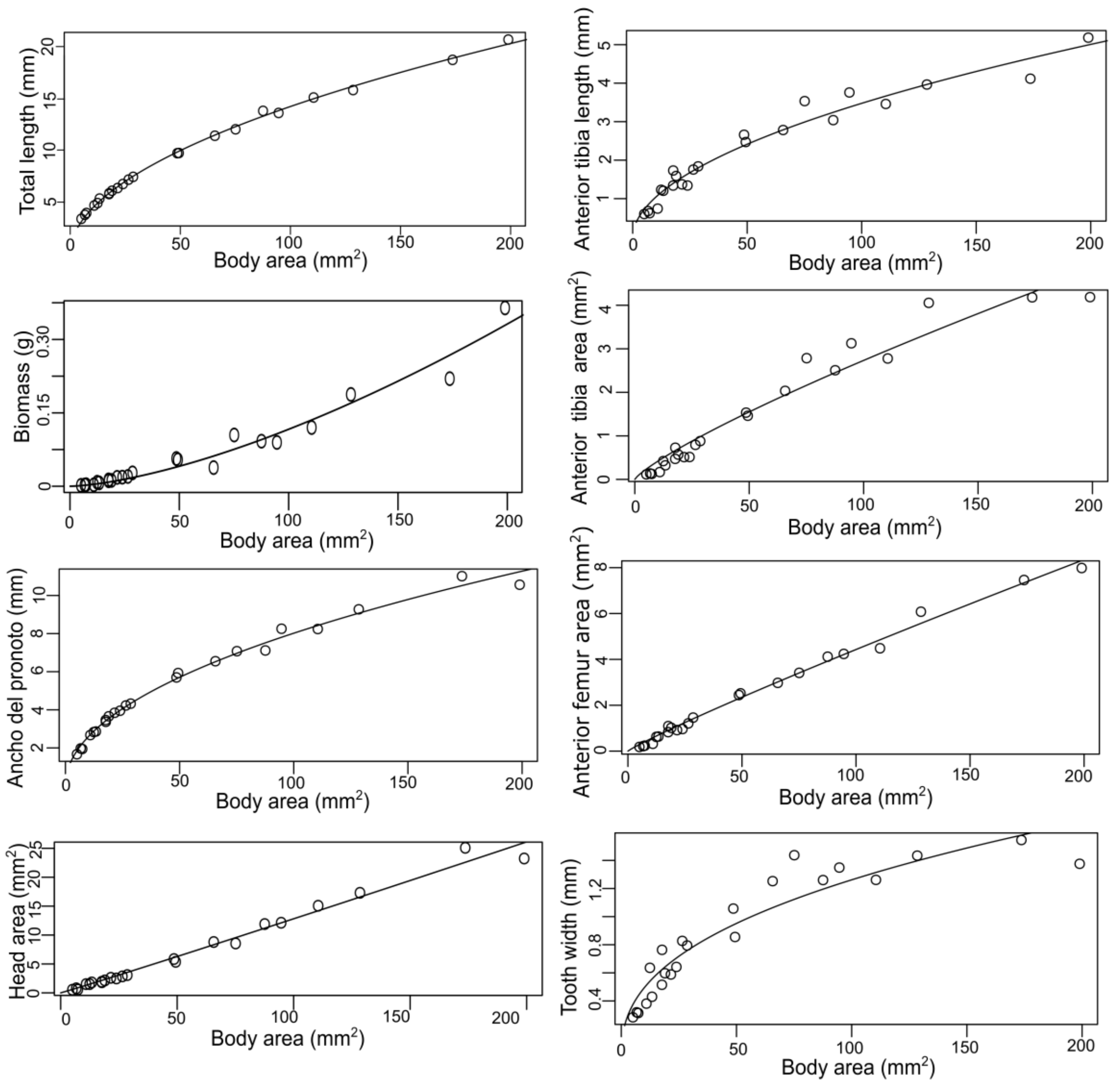

Figure S6.3: Allometric relationships of morphometric traits of female of the Dry Chaco dung beetles.

Table S6.1: Allometric relations between body area and biomass and other morphological variables of Atlantic Forest dung beetles. (* P < 0.05). t: the value of statistics.

	Parameters	Estimators	Standard Error	t
**Total length**	b	0.485	0.010	48.41*
	a	1.625	0.083	19.68*
**Biomass**	b	1.466	0.166	8.838*
	a	0.00009	0.00008	1.091
**Pronotum width**	b	0.517	0.020	25.44*
	a	0.716	0.074	9.65*
**Head area**	b	0.993	0.058	17.23*

	a	0.137	0.042	3.218*
**Anterior tibia length**	b	0.518	0.016	32.12*
	a	0.309	0.025	12.15*
**Anterior tibia area**	b	1.098	0.109	10.036*
	a	0.016	0.009	1.684
**Tooth width**	b	0.469	0.063	7.479*
	a	0.141	0.045	3.154*
**Anterior femur area**	b	0.966	0.041	23.545*
	a	0.054	0.012	4.529*
**Sphericity**	b	-0.003	0.016	-0.213
	a	0.309	0.021	14.872*

Table S6.2: Allometric relations between body area and biomass and other morphological variables of Humid Chaco dung beetles. (* $P < 0.05$). t: the value of statistics.

	**Parameters**	**Estimators**	**Standard Error**	**t**
**Total length**	b	0.492	0.008	60.79*
	a	1.549	0.058	26.66*
**Biomass**	b	1.432	0.038	37.431*
	a	0.00012	0.00002	4.844*
**Pronotum width**	b	0.520	0.014	36.02*
	a	0.731	0.049	14.85*
**Head area**	b	0.988	0.022	45.601*
	a	0.129	0.014	8.958*
**Anterior tibia length**	b	0.521	0.026	20.248*
	a	0.319	0.038	8.315*
**Anterior tibia área**	b	1.204	0.082	14.717*
	a	0.011	0.005	2.312*
**Tooth width**	b	0.535	0.045	11.94*
	a	0.112	0.024	4.756*
**Anterior femur area**	b	1.013	0.037	27.063*
	a	0.045	0.009	5.165*
**Sphericity**	b	0.004	0.015	0.262
	a	0.296	0.017	17.873*

Table S6.3: Allometric relations between body area and biomass and other morphological variables of Dry Chaco dung beetles. (* $P < 0.05$). t: the value of statistics.

	Parameters	Estimators	Standard Error	t
**Total length**	b	0.516	0.006	93.27*
	a	1.379	0.034	39.99*
**Biomass**	b	1.510	0.101	15.008*
	a	0.00007	0.00004	1.967*
**Pronotum width**	b	0.491	0.011	44.61*
	a	0.835	0.041	20.21*
**Head area**	b	1.027	0.040	25.668*
	a	0.113	0.022	5.099*
**Anterior tibia length**	b	0.526	0.026	19.899*
	a	0.308	0.037	8.339*
**Anterior tibia area**	b	0.818	0.054	15.216*
	a	0.063	0.016	3.883*
**Tooth width**	b	0.407	0.034	12.03*
	a	0.194	0.029	6.748*
**Anterior femur area**	b	0.915	0.024	38.625*
	a	0.066	0.008	8.716*
**Sphericity**	b	-0.013	0.018	-0.744
	a	0.340	0.022	15.506*

Appendix S7: Analysis of the principal components of climate, vegetation, and microclimatic conditions.

Eigen-values and their contribution to correlations

Importance of components:

Climate

	PC1	PC2	PC3
Standard deviation	1.15	0.73	0.21
Eigenvalue	2.408	0.5472	0.04482

Explained proportion	0.8027	0.1824	0.01494
Accumulated proportion	0.8027	0.9851	1

Eigenvectors scaled to unit length

	PC1	PC2	PC3
Average annual temperature °C	0.5427072	0.71823249	0.4354434
Mean Diurnal Range °C	0.5489138	-0.69569181	0.4633643
Seasonality of precipitation	0.6357377	-0.01245023	-0.7718047

Microclimatic conditions

	PC1	PC2	PC3	PC4
Standard deviation	1.6351	1.0678	0.3858	0.1931
Eigenvalue	2.6736	1.1402	0.14886	0.037273
Explained proportion	0.6684	0.2851	0.03721	0.009318
Accumulated proportion	0.6684	0.9535	0.99068	1

Eigenvectors scaled to unit length

	PC1	PC2	PC3	PC4
Thermal amplitude	0.5885704	-0.1705617	0.3944965	-0.6847379
Relative humidity %	-0.5187645	0.4183134	0.7347219	-0.1268113
Average temperature °C	0.2168475	0.8635614	-0.3814446	-0.2484732
Maximum temperature °C	0.5809005	0.2240187	0.3988186	0.6732859

Vegetation

	PC1	PC2	PC3	PC4	PC5	PC6
Standard deviation	1.8762	1.0653	0.8442	0.57561	0.49229	0.2427
Eigenvalue	3.52	1.1348	0.7126	0.33132	0.24235	0.058905
Explained proportion	0.5867	0.1891	0.1188	0.05522	0.04039	0.009817
Accumulated proportion	0.5867	0.7758	0.8946	0.94979	0.99018	1

Eigenvectors scaled to unit length

	PC1	PC2	PC3	PC4	PC5	PC6
Bare soil	-0.2321943	-0.72024913	0.4751063	0.36491312	0.26140178	0.01041998
Canopy	-0.496174	0.15697346	-0.232419	0.04443209	0.38114489	-0.72657175
Shrubs	-0.4374312	-0.30173949	0.1181602	-0.70277162	-0.45032175	-0.08347316
Herbs	-0.2262312	0.58872765	0.7720794	0.05320538	-0.05669354	0.00822261
